# Impact of a novel molecular TB diagnostic system in patients at high risk of TB mortality in rural South Africa (Uchwepheshe): study protocol for a cluster randomised trial

**DOI:** 10.1186/1745-6215-14-170

**Published:** 2013-06-12

**Authors:** Richard J Lessells, Graham S Cooke, Nuala McGrath, Mark P Nicol, Marie-Louise Newell, Peter Godfrey-Faussett

**Affiliations:** 1 Department of Clinical Research, London School of Hygiene and Tropical Medicine, Keppel Street, London WC1E 7HT, UK; 2 Africa Centre for Health and Population Studies, University of KwaZulu-Natal, Mtubatuba, South Africa; 3 Department of Infectious Disease, Imperial College, London, UK; 4 Academic Unit of Primary Care and Population Sciences and Academic Unit of Social Sciences, University of Southampton, Southampton, UK; 5 Division of Medical Microbiology, University of Cape Town, Cape Town, South Africa; 6 UCL Institute of Child Health, London, UK

**Keywords:** Tuberculosis, Multidrug-resistant tuberculosis, HIV, Molecular diagnostics, Point-of-care systems, Clinical trial

## Abstract

**Background:**

Tuberculosis control in sub-Saharan Africa has long been hampered by poor diagnostics and weak health systems. New molecular diagnostics, such as the Xpert® MTB/RIF assay, have the potential to improve patient outcomes. We present a cluster randomised trial designed to evaluate whether the positioning of this diagnostic system within the health system has an impact on important patient-level outcomes.

**Methods/Design:**

This pragmatic cluster randomised clinical trial compared two positioning strategies for the Xpert MTB/RIF system: centralised laboratory versus primary health care clinic. The cluster (unit of randomisation) is a 2-week time block at the trial clinic. Adult pulmonary tuberculosis suspects with confirmed human immunodeficiency virus infection and/or at high risk of multidrug-resistant tuberculosis are enrolled from the primary health care clinic. The primary outcome measure is the proportion of culture-confirmed pulmonary tuberculosis cases initiated on appropriate treatment within 30 days of initial clinic visit. Univariate logistic regression will be performed as the primary analysis using generalised estimating equations with a binomial distribution function and a logit link.

**Conclusion:**

Diagnostic research tends to focus only on performance of diagnostic tests rather than on patient-important outcomes. This trial has been designed to improve the quality of evidence around diagnostic strategies and to inform the scale-up of new tuberculosis diagnostics within public health systems in high-burden settings.

**Trial registration:**

Current Controlled Trials ISRCTN18642314; South African National Clinical Trials Registry DOH-27-0711-3568.

## Background

Control of the tuberculosis (TB) epidemic in sub-Saharan Africa is a major public health challenge [1,2]. The epidemic has been exacerbated by the co-existent explosive human immunodeficiency virus (HIV) epidemic and the emergence of drug-resistant *Mycobacterium tuberculosis* strains leading to high mortality rates [2,3]. Enshrined in Millennium Development Goal 6 and the Stop TB Partnership Global Plan 2006–2015 are the targets to reduce TB prevalence and TB mortality rates by 50% (compared to 1990) by 2015 and to eliminate TB as a public health problem by 2050 [4,5]. At current rates of progress these targets will not be achieved in sub-Saharan Africa. New interventions and improved strategies for delivery of interventions are urgently required.

TB control at present relies primarily on the diagnosis and treatment of individuals with active TB disease. Early case detection and initiation of appropriate antituberculous therapy is necessary not only to reduce mortality but also to interrupt transmission. TB microscopy (still the most common diagnostic method in use worldwide) is poorly equipped to control the current TB epidemic in sub-Saharan Africa given its poor sensitivity, particularly in HIV co-infection, and inability to detect drug resistance [6]. Additionally, the placement of diagnostics in centralised facilities distant from where patients seek care contributes to significant delays [7,8] and default [9-13] before initiation of treatment. The impact of this is illustrated most starkly in multidrug-resistant TB (MDR-TB), where delays in culture and drug susceptibility testing (DST) techniques mean that 50% of patients have died by the time their culture/DST result is available [14,15].

The development of novel molecular tools, in particular the Xpert® MTB/RIF assay, offers new opportunities to tackle these problems. This is a fully automated, closed cartridge diagnostic system that utilises hemi-nested polymerase chain reaction (PCR) and molecular beacon technology to detect the presence of *Mycobacterium tuberculosis* and rifampicin-resistant mutations directly from clinical samples in less than 2 h [16-18]. The World Health Organization (WHO) recommended the system be implemented in high-burden settings on the basis of initial data from validation and demonstration studies [19-21]. Many countries are now moving ahead with implementation and there is a need for research to address key questions in the early phase of implementation so as to inform future scale-up [21]. One critical question relates to the optimal positioning of the diagnostic system within different health systems, and this is the focus of the research study.

The primary objective is to test the hypothesis that timely initiation of appropriate TB treatment when the diagnostic system is positioned at the primary health care clinic (point of care) is different from when the diagnostic system is positioned centrally at the district hospital laboratory. Secondary objectives are:

•To evaluate the impact of Xpert MTB/RIF positioning on additional clinical outcomes (mortality, hospital admission, time to initiation of antiretroviral therapy)

•To explore the cost-effectiveness of Xpert MTB/RIF implementation at primary health care clinic level

•To compare the operational feasibility of Xpert MTB/RIF placement at the primary health care clinic level and district hospital laboratory level.

## Methods/Design

### Setting

**Figure 1:**
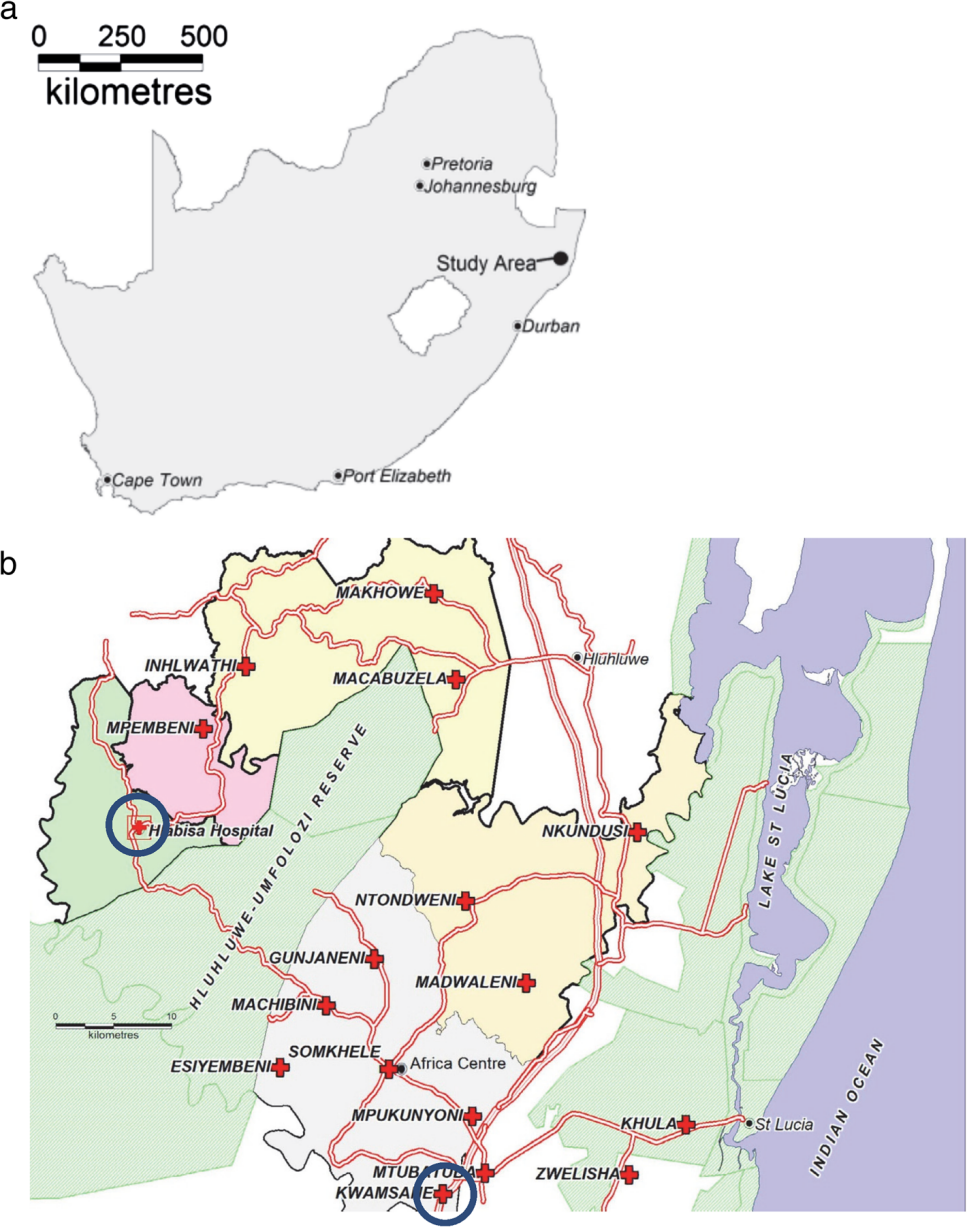
Maps showing location of (a) the study site and (b) primary health care clinic (trial clinic) and district hospital.

The trial is being conducted in Hlabisa health sub-district, uMkhanyakude district, northern KwaZulu-Natal, South Africa (Figure 1). This area has an extremely high dual burden of TB and HIV: the TB notification rate for the sub-district in 2010 was 1,130/100,000; HIV seroprevalence in the adult population (≥15 years) within the Africa Centre surveillance area was 24.1% in 2010; in 2008, 76% of TB cases were associated with HIV infection [22]. In the years 2000–2006 HIV and TB accounted for 71.5% of deaths in young adults (25–49 years) in the Africa Centre surveillance area [23]. HIV and TB treatment and care are delivered at 17 primary health care (PHC) clinics through decentralised collaborative programmes. Participants are recruited from the largest PHC clinic that is situated within a small urban township in the south of the sub-district, approximately 60 km by road from the district hospital.

### Study design

**Figure 2:**
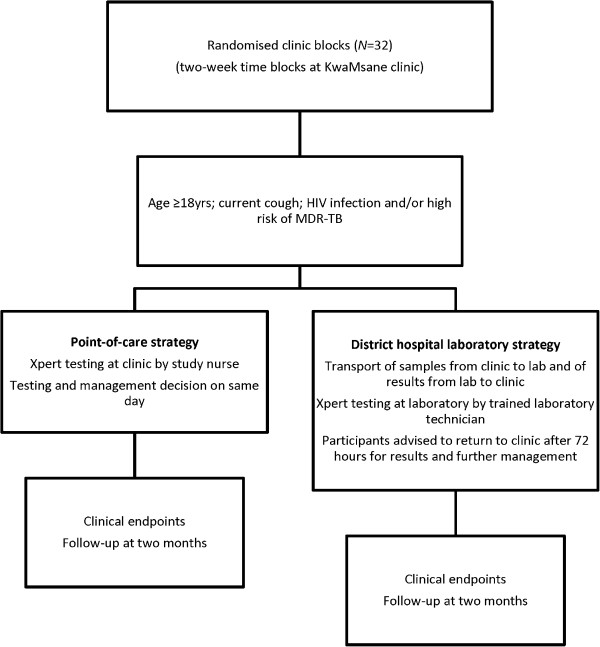
Trial schema.

The study is a pragmatic cluster randomised clinical trial comparing two positioning strategies for the Xpert MTB/RIF system: positioning at centralised laboratory level (district hospital laboratory) versus positioning at primary health care clinic level (point of care). The cluster (unit of randomisation) is a 2-week time block at the primary health care clinic (clinic blocks), and clusters are randomly assigned to the district hospital laboratory strategy or point-of-care strategy. The trial schema is shown in Figure 2.

### Participants

Adult (≥18 years) pulmonary TB suspects with confirmed HIV infection and/or at high risk of MDR-TB are included after giving informed consent. These criteria were defined because of the high risk for mortality in these groups and prioritisation for Xpert MTB/RIF testing, in line with the WHO recommendations [20]. A TB suspect is defined for the purposes of the trial as an individual with a current cough (of any duration) with or without other symptoms. High risk of MDR-TB is defined according to national and international guidelines and incorporates the following categories: failure of the standard treatment regimen (2HRZE/4HR), failure of the re-treatment regimen (2HRZES/1HRZE/5HRE), acid-fast bacilli (AFB) smear non-conversion at month 2 or 3 of the standard or re-treatment regimen, relapse or return after default, any other previous TB (4 or more weeks of TB treatment), household contact with a known MDR-TB case, prison inmate within the last 12 months and health care worker [24,25]. Participants are excluded if they report a previous diagnosis of MDR-TB or extensively drug-resistant TB (XDR-TB), are severely unwell requiring admission to hospital, or are unable to give informed consent. Participants are recruited between the times of 0800 and 1630, Monday to Friday.

### Interventions

All participants provide two spontaneously expectorated sputum specimens on the day of enrolment (spot specimens). The first sputum specimen is submitted for Xpert MTB/RIF testing. The second specimen is submitted for Mycobacterial Growth Indicator Tube (MGIT) culture, line probe assay (LPA) ± phenotypic drug susceptibility testing (DST). In both strategies, the specimen for culture/LPA/DST is transported daily (in the afternoon) to the National Health Laboratory Service (NHLS) laboratory at the district hospital and then onwards to the provincial NHLS referral laboratory. The results of this are used to define TB cases and to define the primary outcome measure.

#### District hospital laboratory strategy

Sputum specimens are transported on a daily basis to the National Health Laboratory Service (NHLS) laboratory at the district hospital using the routine sample transport system. Xpert MTB/RIF testing is performed by a trained laboratory technician at the earliest convenience (within 24 h of the specimen being received in the laboratory) and printed results are returned to the clinic using the same routine transport system. Under this strategy, participants are requested to return for results after 72 h.

#### Point-of-care (POC) strategy

**Figure 3:**
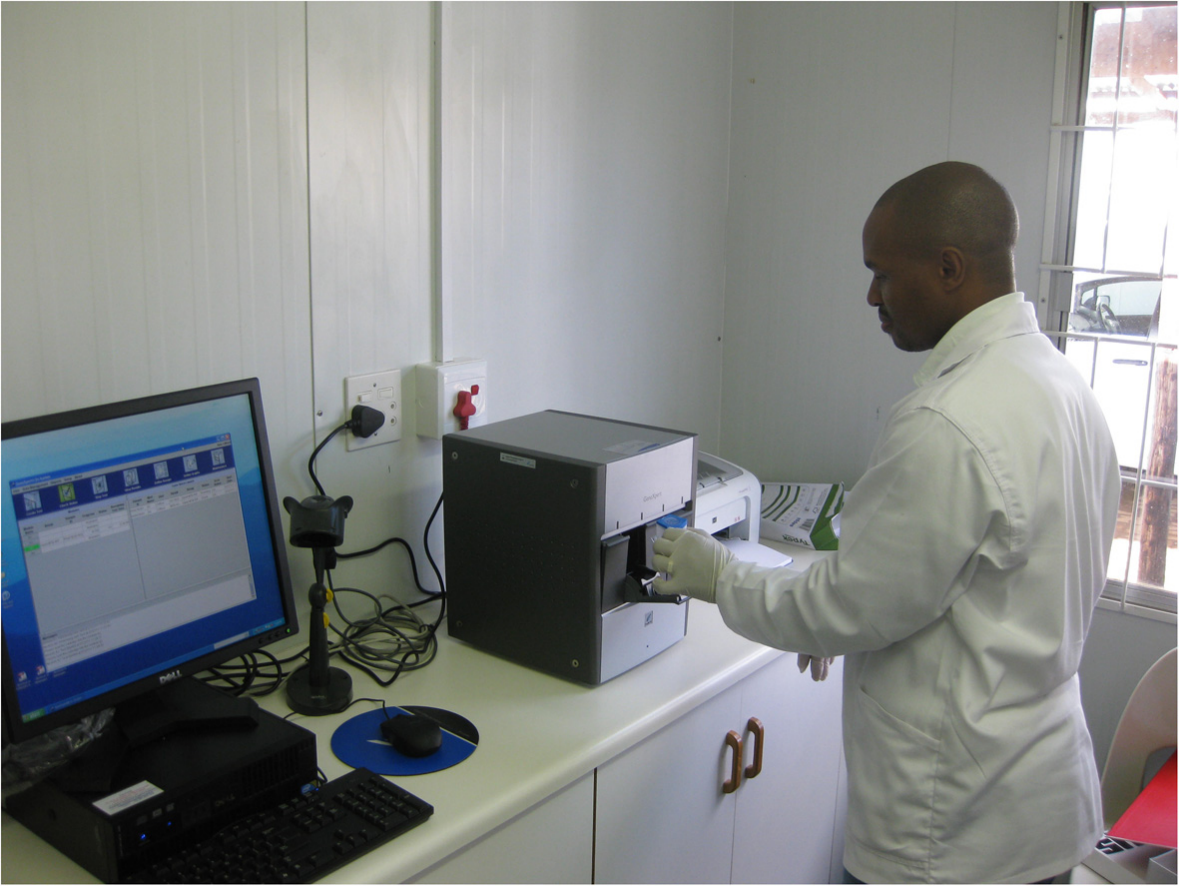
Professional nurse operating the Xpert MTB/RIF system at the primary health care clinic.

The diagnostic system is located at the primary health care in a dedicated room close to the TB clinic (Figure 3). Xpert MTB/RIF testing is performed immediately by the study nurse, on the same day where possible. Participants are invited to wait for the result (approximately 2 h) or, if they are unable to wait or it is towards the end of the working day, they are advised to return the following day.

### Outcome measures

The observational unit for all analyses is the individual participant. The primary outcome for the study is the proportion of culture-confirmed pulmonary TB cases initiated on appropriate TB treatment within 30 days of initial clinic visit (appropriate treatment defined according to results of LPA ± phenotypic DST on the culture isolate).

Secondary outcomes at an individual level are the following, with all time-to-event analyses using the initial clinic visit as time zero:

•All-cause mortality in TB suspects and MDR-TB suspects at 60 days

•Time to initiation of appropriate TB treatment (days) for culture-confirmed pulmonary TB cases

•Time to initiation of MDR-TB treatment (for MDR-TB cases confirmed by culture/LPA/DST)

•Proportion of TB suspects and MDR-TB suspects with at least one hospital attendance within 60 days

•Time to initiation of antiretroviral therapy (ART) for HIV-infected TB suspects and MDR-TB suspects not yet receiving but eligible for ART

•Sensitivity and specificity of Xpert MTB/RIF

▪ for *M. tuberculosis* detection (compared to reference standard of single MGIT culture)

▪ for detection of rifampicin resistance (compared to reference standard of phenotypic DST ± LPA)

### Sample size

The study was designed to detect an increase from 85% to 95% in the proportion of culture-confirmed pulmonary TB cases initiated on appropriate treatment within 30 days. Sample size was calculated with the equation of Hayes and Bennett, using the coefficient of variation (κ) [26]. With κ = 0.05 and a cluster size of 12 culture-positive cases, we would need 16 clusters and 188 culture-positive TB cases in each arm to detect this difference with 95% confidence and 80% power. We assumed 10% of individual participants would be lost to follow-up at 60 days, so we would need 208 culture-positive TB cases in each arm. Based on the assumption that 25% of TB suspects would have a positive MGIT culture, we would require enrolment of 1,664 TB suspects. The total sample size will therefore be 32 clusters and 1,664 individual participants.

The coefficient of variation (κ) is small, but as the clusters are clinic time blocks rather than geographic areas or health care facilities, minimal variation is expected between clusters. This value of κ corresponds to a range of proportions appropriately treated within 30 days in the district hospital laboratory arm of 77-94%.

For the secondary endpoint of all-cause mortality within 60 days, the analysis will incorporate all participants (all suspects), regardless of presence or absence of TB disease. The sample size of 32 clusters and 60 participants per cluster gives approximately 80% power to detect a 33% reduction in mortality from 12% in the district hospital laboratory arm to 8% in the point-of-care arm, with 95% confidence.

### Randomisation

The allocation schedule for random assignment of 2-week time blocks was computer generated, using random permuted blocks of eight. Allocation for each clinic block was placed into sealed envelopes by the statistician; the principal investigator opens the envelope on the Friday before the start of a new 2-week block and communicates the allocation for the next 2 weeks to study staff.

### Implementation

Health care workers at the primary health care clinic identify potential participants. All individuals reporting cough are referred to the study nurse. Eligibility criteria are checked by the nurse, and subjects meeting the inclusion criteria are provided spoken and written information about the study in isiZulu and/or English; those willing to participate are taken through the informed consent process and are asked to provide a signature or thumbprint on the consent form.

A baseline assessment is performed by the study nurse. Demographic information, current symptoms, previous TB history, risk factors for drug resistance, HIV status, and history of ART use are documented on a case report form.

With both strategies, clinical decisions are made by the study nurse on the basis of the Xpert MTB/RIF result and according to pre-defined algorithms. TB patients without resistance to rifampicin are commenced on standard anti-TB therapy (4HRZE/2HR) by the study nurse. All patients with rifampicin-resistant TB are reported to the trial physician on the same day and are subsequently referred to the specialist drug-resistant TB treatment centre in Durban. Management of suspects with a negative Xpert MTB/RIF follows existing protocols for smear-negative TB suspects: oral antibiotics are prescribed and patients are advised to return if symptoms do not improve after 14 days. Patients who remain symptomatic following this course of antibiotics can be referred to the district hospital for chest X-ray and physician review. Results from MGIT culture and DST are returned through the routine laboratory system and are used to guide clinical management where appropriate.

### Outcome evaluation

To ascertain the primary and secondary outcomes, at enrolment all participants are allocated a review date 2 months from the enrolment visit. Participants are invited to attend clinic for review but are also invited to consent to telephonic follow-up and/or home visit in case clinic visit is not possible. Additional contact details are provided for at least one other family member (or other person designated by participant) at enrolment, wherever possible. Participants are told that, when attending the clinic for the follow-up visit, they will be reimbursed with a ZAR 50 grocery voucher (approximately equivalent to USD 6). Outcome data pertaining to TB treatment initiation, additional investigations, hospital attendances and admissions, and ART initiation (where appropriate) are collected on a case report form by the study nurse. In the event that no contact is made with patient or with named contact persons, follow-up information is collected from the clinic TB registers and the operational HIV programme database – permission to use these data is also included in the informed consent process.

### Statistical analysis

Analysis of baseline characteristics will be performed to characterise the study population and to identify baseline imbalances between the study arms in order to decide whether any covariates need to be adjusted for in the final analyses. The baseline data will include: age, sex, body mass index (BMI), history of previous TB, HIV infection status, CD4+ cell count, and use of antiretroviral therapy and isoniazid preventive therapy.

All final analyses will be intention-to-treat analyses performed at the individual level taking account of within-cluster correlation. The definition of TB cases for the primary outcome will be based on MGIT culture positivity. The proportion of TB cases initiated on appropriate TB treatment within 30 days will be based on whether the appropriate treatment regimen was commenced within 30 days of the initial clinic visit—appropriate regimens are defined according to drug susceptibility pattern and with reference to national guidelines (Table 1) [27]. The primary analysis will include only TB cases not on TB treatment at the time of enrolment, i.e. excluding smear non-converters or failures still on treatment. The primary outcome is a binary variable (initiation of appropriate treatment or not) so univariate logistic regression will be performed as the primary analysis using generalised estimating equations (GEE) with a binomial distribution function and a logit link [28]. The odds ratio will be reported with 95% confidence intervals and a *p*-value from the Wald test. This method will allow for the correlation between observations (within clusters) without needing to specify a distributional assumption for the correlations. In addition, important individual-level characteristics that are unbalanced between arms will be included in the model as covariates. For the secondary outcomes with binary variables, GEE models will also be fitted with a binomial distribution function and a logit link. For the secondary outcomes with time-to-event measures, Cox proportional hazard models will be fitted with the shared frailty option to account for the cluster randomisation [29]. Hazard ratios will be presented with 95% confidence intervals.

**Table 1:** Definitions of appropriate initial anti-TB drug regimen for primary outcome measurement

Case definition*	**Appropriate initial anti-TB drug regimen**
*M. tuberculosis* susceptible to rifampicin and isoniazid	Isoniazid + rifampicin + pyrazinamide + ethambutol
*M. tuberculosis* with mono-resistance to isoniazid	Isoniazid + rifampicin + pyrazinamide + ethambutol
*M. tuberculosis* with mono-resistance to rifampicin	Standardised second-line regimen§ with isoniazid
Multidrug-resistant *M. tuberculosis* (MDR-TB)†	Standardised second-line regimen§
Extensively drug-resistant *M. tuberculosis* (XDR-TB)†	Standardised XDR-TB regimenǁ

* Case definition based on results of MGIT culture + line probe assay + phenotypic DST.

† MDR-TB defined as resistance to rifampicin and isoniazid.

† XDR-TB defined as MDR plus resistance to ofloxacin and kanamycin.

§Standardised regimen according to national guidelines (kanamycin/amikacin + fluoroquinolone + ethionamide + cycloserine/terizidone ± pyrazinamide ± ethambutol) [27].

ǁStandardised regimen according to national guidelines (capreomycin + fluoroquinolone + ethionamide + cycloserine/terizidone + PAS + clofazimine) [27].

The diagnostic performance of Xpert MTB/RIF will be compared between the two arms. Estimation of sensitivity and specificity of Xpert MTB/RIF for the detection of *M. tuberculosis* against the reference standard of single MGIT culture will be based on complete case analysis (participants with paired valid Xpert MTB/RIF and MGIT culture results) and will only include individuals not on TB treatment at the time of enrolment. Estimation of sensitivity and specificity of Xpert MTB/RIF for the detection of rifampicin resistance against the reference standard of genotypic ± phenotypic DST on the culture isolate will be based on participants with *M. tuberculosis* detected by Xpert MTB/RIF and with a positive MGIT culture and valid drug susceptibility test (LPA ± phenotypic DST) results. This will include individuals on TB treatment at the time of enrolment (e.g. participants with AFB smear non-conversion or treatment failure).

### Economic evaluation

In addition to evaluating the effectiveness of point-of-care positioning of Xpert MTB/RIF, data from the trial will be combined with those from a costing analysis to explore the cost-effectiveness of point-of-care placement. Health system costs will be obtained through monitoring of study expenditure and interviews with health service management. Collection of data relating to patient and household costs will be nested within the trial—this will involve additional data collected from a subset of patients at baseline and at the 2-month follow-up to determine direct and indirect costs incurred during the diagnostic process. The framework for costing analysis is presented in Table 2. The health system costs and patient costs will be combined with the outcome data to generate an average incremental cost per TB case appropriately treated.

**Table 2:** Components of cost analysis

**Health service costs**	**Patient and household costs**
**Direct costs associated with diagnostic services**	**Direct costs**
***Fixed***	***Transport***
Building space	Transport to/from clinic (patient ± carer)
Utilities	Transport to/from hospital (patient ± carer)
GeneXpert machine	***Non-transport***
Staff training	Medication
Internal/external QC	OPD attendance
GeneXpert calibration	X-rays
***Variable***	GP consultation
Xpert MTB/RIF tests	Traditional healer consultation
Consumables (gloves, N95 masks)	
Specimen transport	
Staff work time (based on time analysis)	
GeneXpert maintenance	
**Direct costs associated with medical services**	**Indirect costs**
First-line TB therapy	Lost time (salary) at work (patient ± carer)
MDR-TB therapy	
Hospital admission	
OPD attendance	
Clinic attendance	

### Operational feasibility

The study will also compare the operational feasibility of Xpert MTB/RIF implementation at the hospital laboratory and at the primary health care clinic. This encompasses an assessment of the performance and robustness of the system, as well as evaluation of the practicality of operating the system at the laboratory and at the clinic. The key indicators to be assessed are displayed in Table 3. Data on these indicators will be collected throughout the trial.

**Table 3:** Indicators for operational feasibility evaluation

**Indicator**	**Method of measurement**
Power supply	Time log for power cuts/generator use
Operating temperature for GeneXpert machine	Temperature log
Storage temperature for Xpert MTB/RIF kits	Temperature log
Hands-on user time	Activity log
Indeterminate results	GeneXpert software
Data errors (incomplete identifiers etc.)	GeneXpert software
Maintenance needs	Requirement for supplier support
Training requirements	Recording of initial and follow-up training sessions
Supervision requirements	Log of assistance from other laboratory staff/PI
Waste management	Recording of problems with disposal of used cartridges
User appraisal	Regular appraisal by laboratory staff and study staff
User performance	Regular independent observation of staff performance

### Ethical considerations

The study has been approved by the Biomedical Research Ethics Committee of the University of KwaZulu-Natal (BF033/11), the Ethics Committee of the London School of Hygiene and Tropical Medicine (5926), and the Health Research Committee of the KwaZulu-Natal Department of Health (HRKM084/11). Permission for the study was granted by Hlabisa Hospital and by the Community Advisory Board of the Africa Centre for Health and Population Studies.

Given that the units of randomisation are time blocks, it is not possible for individuals to consent to randomisation. Individual consent for participation remains important given that the intervention is delivered to individuals and that individual-level data are collected at enrolment and at follow-up.

There are TB suspects who are not eligible for this study and therefore will not have access to Xpert MTB/RIF testing within the study (suspects who are neither HIV-infected nor at high risk for MDR-TB). The justification for this is that these groups were a lower priority for this intervention given the much lower mortality rates and these suspects continue to receive diagnostic evaluation including sputum microscopy ± culture/LPA/DST according to national guidelines. At the time of study design it was predicted that, were Xpert MTB/RIF to be implemented in South Africa, the WHO recommendations would be followed (use in HIV-infected and those at high risk of MDR-TB). Although the national roll-out plan went beyond this in incorporating its use for all TB suspects, Xpert MTB/RIF has not yet been installed in Hlabisa sub-district and is therefore not yet available in the sub-district outside the trial.

## Discussion

Evaluation of diagnostic tools provides different challenges than those of therapeutic interventions. Diagnostic accuracy studies are usually the starting point for evaluation of new technologies, yet to inform public health policies and implementation it is crucial to evaluate patient-important outcomes [30]. The ultimate impact of a new diagnostic test should be measured by its capacity to generate beneficial outcomes. A randomised trial is the most rigorous design to evaluate clinical outcomes from a diagnostic intervention. Individual randomisation was considered logistically challenging and potentially disruptive in the context of a busy clinic and laboratory system, although this would have been the most efficient statistical design [28]. Cluster randomisation by health care facility was not possible given the limited resources. Therefore cluster randomisation by time block was the preferred study design to maximise internal validity and to minimise disruption to clinic services. Other trial designs were considered (non-randomised controlled trial with allocation by day/week/month or controlled before and after study) but were felt to be inferior in addressing the research hypothesis, mainly because of the potential for bias and therefore loss of internal validity. There have been few published trials where the unit of randomisation is a time block rather than a geographical or organisational cluster. The trials that have been published have not used consistent methods for sample size calculation—some have adjusted appropriately for cluster variation [31-33] whereas others have arbitrarily inflated the sample size from that for an individual RCT [34] and others have based the sample size on the numbers available to participate [35].

Blinding of patients or of health care workers is not feasible in this pragmatic diagnostic trial because allocation to trial arms involves different actions by the patient and the clinical staff. As a result of this, the outcomes are as objective as possible to limit potential bias from differential ascertainment of outcomes in the two arms. The possibility of differential recruitment into the trial arms exists but will be minimised by standardised referral criteria for the clinic health care workers; recruitment will be monitored by reviewing the clinic records to ascertain what proportion of patients with cough were referred to the study during each 2-week block. This will be reported if there is a major imbalance in recruitment to the two trial arms. There is a further risk of selection bias if there is differential non-participation. The proportions of eligible subjects consenting by trial arm will be monitored and will be reported accurately at the conclusion of the trial. There is some risk of contamination between the arms if, over time with point-of-care testing, the health workers see the importance and the effect of receiving the test result in a timely fashion and this then improves their ability to encourage all suspects to return and receive their result. This would tend to bias the findings towards the null hypothesis. We will explore this by assessment of the variability in the proportion returning for their test result by cluster and by time period.

The evaluation of diagnostic accuracy is not the primary focus of the trial and the reference standard of a single culture could be considered an imperfect gold standard. In the initial Xpert MTB/RIF clinical validity studies, the reference standard used results of liquid and solid culture on two specimens (four culture results in total) [17]. Conversely, in the later demonstration studies, the reference standard varied between study sites and in some sites included results of only a single culture [18]. Observational data from the district in 2007 suggested that 5% of all culture-positive cases were multidrug-resistant [36]. Given that we will preferentially include suspects with a high risk of MDR-TB, we expect the overall proportion with MDR-TB to be at least 10%. It is possible that the impact of Xpert MTB/RIF positioning may be different for the drug-susceptible and drug-resistant cases. If this is the case then a higher or lower than expected proportion with MDR-TB could modify the effect of point-of-care placement, and this will be explored in secondary analyses.

There are a number of trials evaluating the impact of Xpert MTB/RIF in different settings and with different research hypotheses. Information about research projects is collated by the TREAT TB Xpert Research Mapping Project [37]. Several studies are examining point-of-care implementation but, to our knowledge, this is the only study directly comparing point-of-care use to centralised laboratory use. There is already some evidence from South Africa of the feasibility of implementation at the primary health care level, although several operational challenges were experienced when implemented within a very large urban clinic [38,39]. This study should provide direct evidence of any benefits of point-of-care positioning as well as further information about the costs and logistical challenges of such strategies. This can also be considered as a proof-of-principle study that will help to understand the benefits of bringing diagnostics closer to patients, and this will have broader relevance as we continue to develop and evaluate diagnostic technologies suitable for point-of-care use [40,41].

## Trial status

The study received final ethical approval in June 2011. Enrolment commenced on 22 August 2011. Enrolment is scheduled to complete in March 2013 and follow-up will be complete in May 2013.

## Abbreviations

ART: Antiretroviral therapy; DST: Drug susceptibility testing; GEE: Generalised estimating equation; HIV: Human immunodeficiency virus; MDR-TB: Multidrug-resistant TB; MGIT: Mycobacterial growth indicator tube; NHLS: National Health Laboratory Service; PCR: Polymerase chain reaction; PHC: Primary health care; TB: Tuberculosis; WHO: World Health Organization; XDR-TB: Extensively drug-resistant TB.

## Competing interests

The authors declare that they have no competing interests.

## Authors’ contributions

RJL and PGF conceived and designed the trial, with additional input from GSC, NM, MPN and MLN. NM helped with the statistical design and analysis plan. RJL wrote the first draft of the manuscript. All authors contributed to revision of the manuscript and approved the final version.
